# Computational aspects underlying genome to phenome analysis in plants

**DOI:** 10.1111/tpj.14179

**Published:** 2019-01-12

**Authors:** Anthony M. Bolger, Hendrik Poorter, Kathryn Dumschott, Marie E. Bolger, Daniel Arend, Sonia Osorio, Heidrun Gundlach, Klaus F. X. Mayer, Matthias Lange, Uwe Scholz, Björn Usadel

**Affiliations:** ^1^ Institute for Biology I, BioSC RWTH Aachen University Worringer Weg 3 52074 Aachen Germany; ^2^ Forschungszentrum Jülich (FZJ) Institute of Bio‐ and Geosciences (IBG‐2) Plant Sciences Wilhelm‐Johnen‐Straße 52428 Jülich Germany; ^3^ Department of Biological Sciences Macquarie University North Ryde NSW 2109 Australia; ^4^ Leibniz Institute of Plant Genetics and Crop Plant Research (IPK) Gatersleben Corrensstraße 3 06466 Seeland Germany; ^5^ Department of Molecular Biology and Biochemistry Instituto de Hortofruticultura Subtropical y Mediterránea “La Mayora” Universidad de Málaga‐Consejo Superior de Investigaciones Científicas Campus de Teatinos 29071 Málaga Spain; ^6^ Plant Genome and Systems Biology (PGSB) Helmholtz Zentrum München (HMGU) Ingolstädter Landstraße 1 85764 Neuherberg Germany

**Keywords:** plant genomes, plant bioinformatics, plant genome annotation, phenotyping

## Abstract

Recent advances in genomics technologies have greatly accelerated the progress in both fundamental plant science and applied breeding research. Concurrently, high‐throughput plant phenotyping is becoming widely adopted in the plant community, promising to alleviate the phenotypic bottleneck. While these technological breakthroughs are significantly accelerating quantitative trait locus (QTL) and causal gene identification, challenges to enable even more sophisticated analyses remain. In particular, care needs to be taken to standardize, describe and conduct experiments robustly while relying on plant physiology expertise. In this article, we review the state of the art regarding genome assembly and the future potential of pangenomics in plant research. We also describe the necessity of standardizing and describing phenotypic studies using the Minimum Information About a Plant Phenotyping Experiment (MIAPPE) standard to enable the reuse and integration of phenotypic data. In addition, we show how deep phenotypic data might yield novel trait−trait correlations and review how to link phenotypic data to genomic data. Finally, we provide perspectives on the golden future of machine learning and their potential in linking phenotypes to genomic features.

## Significance statement

This review addresses the state of the art on plant genome sequencing, phenotyping and how to bridge these. Genome sequencing sees a new revolution and phenotyping is becoming extremely important.

## Introduction

The last decade has seen a considerable increase in published plant genomes, enabled by advances in sequencing technologies. The initial post‐Sanger sequencing advancement came in the form of high‐throughput short‐read technologies, frequently termed second‐generation sequencing (see glossary Table 1). Although the maximum read length of about 600 bases was considerably shorter than that available from contemporary Sanger sequencing, the high throughput and low relative cost ensured that this technology was quickly adapted. This was followed by the more recent long‐read technology (third‐generation sequencing) led by the PacBio platform. This overcame the read length problem inherent in second‐generation sequencing, enabling multi‐kilobase reads, but at a cost of read quality. Third‐generation sequencing was initially used to resequence well studied model species such as *Arabidopsis thaliana*, yeast and *Drosophila* (Berlin *et al*., 2015) before successfully sequencing new genomes, such as the small genome from *Oropetium thomaeum* (Van Buren *et al*., 2015).

**Table 1:** Glossary table

Term	Definition
Best linear unbiased predictions (BLUP)	A method used to estimate the ‘random’ effects of a mixed model. For a plant researcher this is of relevance when genotypes are considered a ‘random’ effect (reviewed in Piepho *et al*., 2008).
Chromosomal pseudomolecules	The largest sequences assembled and ordered by genome sequencing projects, each representing a single chromosome in the genome. These are not necessarily complete, i.e. they might contain stretches of ‘N's.
Contigs	Assembled sequences that contain no unknown (‘N’) bases.
Copy‐number variation (CNV)	An InDel that increases or decreases the number of copies of a specific DNA sequence.
De Bruijn graph method	A method of genome assembly particularly suited to datasets from short‐read sequencing platforms, due to its scalability to large numbers of reads.
*De novo* assembly	The method of assembling a genome from scratch when there is no reference sequence available.
Genome‐wide association studies (GWAS)	An observational study that tries to associate a genome‐wide set of variants (e.g. markers/polymorphisms) to determine whether a variant is associated with a particular trait. Usually requires many genotypes and relies on natural populations and/or panels with diverse cultivars as opposed to biparental populations.
Insertions/deletions (InDel)	A genomic variant in which one or more bases have been added and/or removed, resulting in a shorter or longer sequence than originally present.
Machine learning	The process of training computers to autonomously extract important information from a data set and identify patterns. Important subfields for a plant researcher include: (i) classification (e.g. is a plant diseased or healthy given an image); (ii) regression (e.g. predict plant biomass from several images); (iii) clustering (e.g. are there subtypes of plants in the experiment based on the measurement)?
Minimum information about plant phenotyping experiment (MIAPPE)’	Presents guidelines and a checklist for describing plant phenotyping experiments so that they are understandable and reproducible.
Ontology	An ontology is extending controlled vocabularies (i.e. fixed lists of terms to be used) by relating these terms to each other. In the simplest case it could describe one term to always imply another term (e.g. if monocot, dicot and plant could represent a controlled vocabulary and the addition of monocot IS_A plant; dicot IS_A plant would start to add relationships towards an ontology).
Overlap‐layout consensus (OLC) method	A method of genome assembly particularly suited to datasets from long‐read sequencing platforms, originally developed for Sanger sequencing data.
Polish	A post‐assembly quality improvement procedure that aims to identify and correct small scale errors.
Quantitative trait locus (QTL)	A region of DNA containing one or more genes which are associated to the expression of a quantitative phenotypic trait.
Reduced representation libraries (RRL)	A protocol to create a sequencing library that aims to contain sequences only from selected subsets of the source genome.
Restriction site associated DNA sequencing (RAD‐seq)	A protocol using restriction enzymes to target specific sequences from a genome for including in a sequencing library.
Second‐generation sequencing/next‐generation sequencing	Usually sequencing by synthesis based, high‐throughput sequencing platforms that can sequence millions of DNA strands in parallel, but compared with Sanger sequencing have a higher error rate and limited read length, e.g. 50–600 bases, depending on the specific instrument used. Some platforms offer a paired‐end mode, whereby both ends of a DNA fragment are sequenced.
Single nucleotide polymorphism (SNP)	A genomic variant consisting of a single nucleotide substituted for an alternative nucleotide.
Third‐generation sequencing	Single‐molecule sequencing platforms that can create multi‐kilobase reads, but which have much higher error rates than Sanger or second‐generation sequencing platforms.
Variable importance prediction	A formalized method to predict the importance of variables in PLS type analyses.

This trend continues as Oxford Nanopores, the latest long‐read technology, becomes more widely available. This technology (Jain *et al*., 2016) has already successfully been used to reconstruct the Arabidopsis genome (Michael *et al*., 2018) as well as the genome of a non‐model wild tomato species (Schmidt *et al*., 2017) and has the added advantage of not requiring large capital investment. Long reads can not only reveal small‐scale variation and presence−absence dynamics in genes, but also large‐scale variation, including rearrangements from for example transposon activity, and can lead to potentially novel insights into a plant species. Additionally, as pangenomic approaches based on multiple‐reference accessions becomes more common, *de novo* sequencing of many lines from each species can be expected (e.g. *Brassica*: Golicz *et al*., 2016, rice including wild relatives: Zhao *et al*., 2018). While genome research is certainly well established and advances in technologies allow for the delivery of data ever more quickly and efficiently, effective algorithms and storage capacity for genome data are becoming serious concerns (Stephens *et al*., 2015).

As with genomic developments, there are promising advances in plant phenotyping technology, such as the use of automated phenotyping machinery (Fiorani and Schurr, 2013) and advanced image analyses (Pound *et al*., 2014, 2017; Tsaftaris *et al*., 2016). This has resulted in unprecedented insights into plant physiology, architecture, and performance. Compared with genomic research, data output produced by established systems in plant phenotyping is still manageable (Coppens *et al*., 2017), although expanding the use of advanced imaging platforms such as hyperspectral cameras by the wider community will likely result in similar storage capacity concerns. As phenotyping equipment costs are still prohibitive for many plant laboratories, new lower cost phenotyping procedures, including the deployment of inexpensive sensors and set ups (Paulus *et al*., 2014) as well as machine‐learning techniques for low‐cost devices, are being developed and researched (Atanbori *et al*., 2018).

Analyses that combine advanced phenotyping and genomic datasets offer great potential for the discovery of novel insights, such as in GWAS (Millet *et al*., 2016, Borevitz *et al*., ibid) or genomic prediction technologies, even within the scope of a single project. Furthermore, machine‐learning and other data science techniques can extract novel insights from meta analyses of multiple datasets. However, there are several obstacles that need to be addressed before this can become widely applicable. This review outlines the current state of the art in genomics, plant phenotyping, and standardization. It explains how these data can be integrated using data science and machine‐learning techniques, and discusses current challenges that are being addressed by the plant science community.

### From sequences to genomes

*De novo* sequencing and assembly of plants is often difficult and tedious (Claros *et al*., 2012). This is largely due to the high repeat content of many plant genomes, with repetitive elements derived from a wide range of sources, including transposons and tandem gene duplications. The situation can be further complicated by the fact that many plants are autopolyploid, or have undergone recent whole genome duplications (Vogel *et al*., 2018). This has often necessitated analyzing diploid relatives (e.g. wild strawberry, Shulaev *et al*., 2011) or using double‐monoploid lines (e.g. Potato Genome sequencing consortium, 2011) rather than a commercially relevant crop line. Even more problematic, plants may be derived from the hybridization of different but related species, giving rise to allopolyploid species such as rapeseed (Chaloub *et al*., 2014), tobacco (Sierro *et al*., 2014) or petunia (Bombarely *et al*., 2016), whose genomes are often tackled by first analyzing the extant parental genomes. This approach has also been applied to sequencing the D parent of the allohexaploid wheat (Luo *et al*., 2017).

While polyploidy forms an obvious problem, repeats and the complications that they cause have been known but not systematically analyzed. Jiao and Schneeberger (2017) investigated this issue in detail by comparing approximately 100 diverse plant and vertebrate genomes. The authors were able to demonstrate higher incidences of repeats in plant genomes, suggesting that some plant genome assemblies will require more advanced approaches to span repetitive regions. Another difficulty of plant genomes is often their sheer size, making costly long‐read sequencing technologies prohibitively expensive, both in terms of sequencing and computation. This was especially challenging for the complex 17 Gbp wheat genome, which consists of three subgenomes (International Wheat Genome Sequencing Consortium, 2014). Therefore, the initial assembly relied on sequencing sorted chromosomes, a difficult wet‐lab technique. Conversely, a whole‐genome shotgun assembly using long‐read data required 880 000 central processing unit (CPU) h to compute, taking more than 6 months, despite being run on a compute cluster (Zimin *et al*., 2017). Finally, many plants are self‐incompatible (Fujii *et al*., 2016) and consequently can be highly heterozygous, adding complexity to the assembly process.

**Figure 1:**
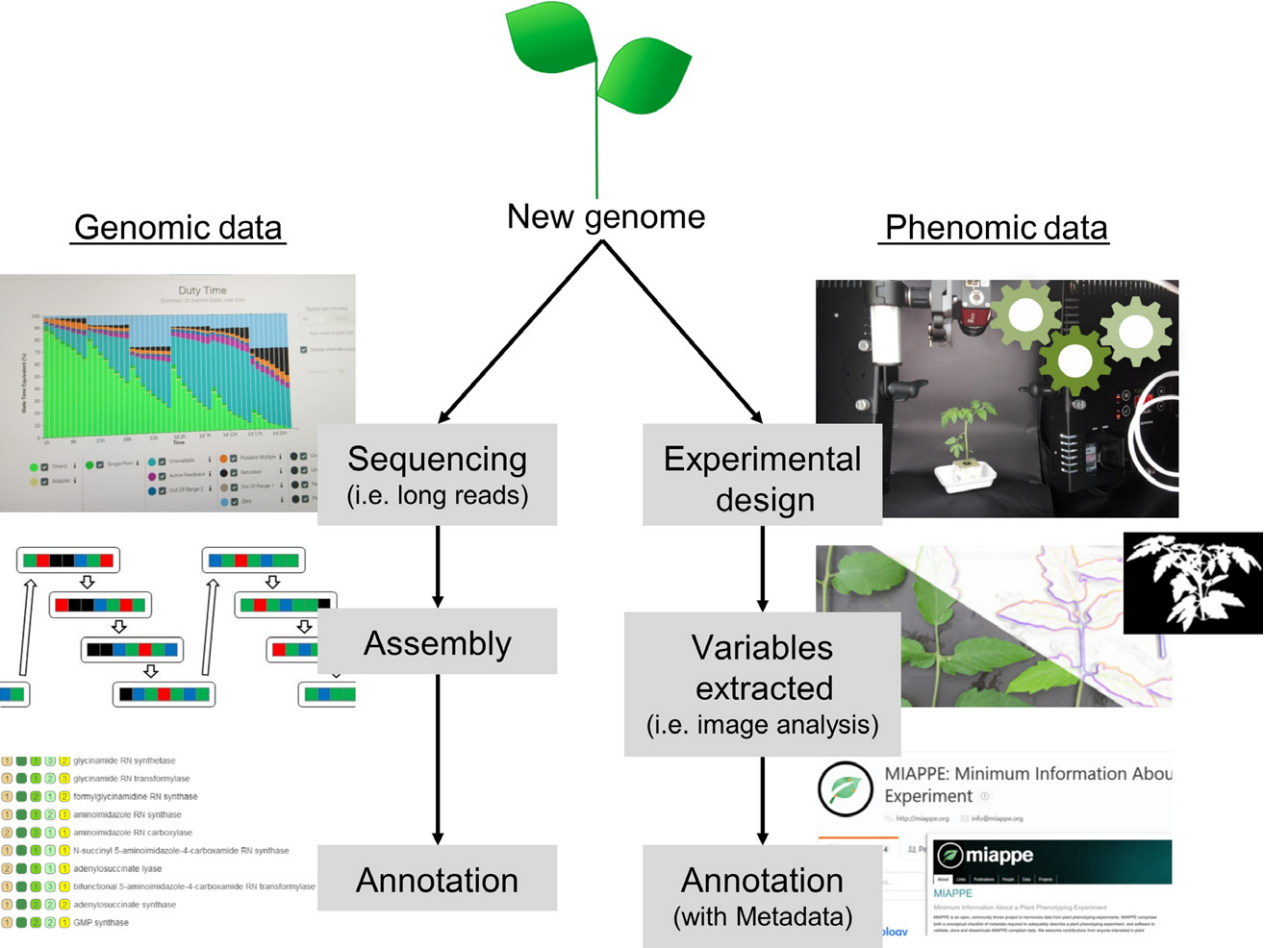
Preparatory analyses for genomics and phenomics data for new genomes.

Despite these hurdles, many standard pipelines and tools that can potentially assemble a reasonable quality genome, are available (Figure 1). The primary factor determining the choice of assembly pipeline is the type of reads in the dataset, as short and long reads are generally assembled using very different approaches. In the case of short‐read data from the Illumina platform, reads are typically quality controlled using for example FASTQC followed by adapter/quality trimming (Bolger *et al*., 2014b). After read trimming, the assembly process can be performed using a variety of short‐read assemblers such as ABySS (Simpson *et al*., 2009), DISCOVAR (*de novo*) (Weisenfeld *et al*., 2014), Velvet (Zerbino and Birney, 2008) or SOAPdenovo (Luo *et al*., 2012). SOAPdenovo is especially popular as it is easy to install, relatively easy to use and reasonably fast. Alternatively, commercial software such as the CLC assembler can be used with small computational resources and offers a graphical user interface, whereas the commercial NRGene suite enables the analysis of complex genomes using short‐read data (Avni *et al*., 2017; Luo *et al*., 2017).

Examples of long‐read assemblers include Miniasm (Li, 2016), Canu (Koren *et al*., 2017), SMART denovo or its successor, wtdbg, and Falcon. In some cases, steps of different assemblers can be ‘mixed and matched’ for speed and efficiency. For instance, it can be beneficial to use the error correction steps of Canu coupled to SMART denovo (Schmidt *et al*., 2017) or wtdbg (Koren: https://genomeinformatics.github.io/na12878update/).

The relative costs and high error rate of long‐read technologies negate some of their benefits. Error rate was particularly problematic for long reads from early versions of the third‐generation Oxford Nanopore platform, which offered a read correctness of below 70% (Rang *et al*., 2018). Its error rate has improved substantially in subsequent versions, but the technology still has difficulty resolving specific base patterns, such as long homopolymers. As a result, recent versions have been assessed to give a maximum read correctness of 88% (Wick *et al*., 2018), although this is potentially lower in plants (Schmidt *et al*., 2017). As these errors are systematic, they cannot be fully corrected by additional coverage. Therefore, even after post‐assembly read polishing, assemblies are currently capped at 99.9% accuracy when using Oxford data alone (Wick *et al*., 2018). In theory, PacBio reads should have much fewer systematic errors, and therefore should converge on the correct result, given sufficient coverage. Nonetheless, there are indications that real‐world assemblies may still suffer from some residual accuracy problems (Watson, 2018).

**Figure 2:**
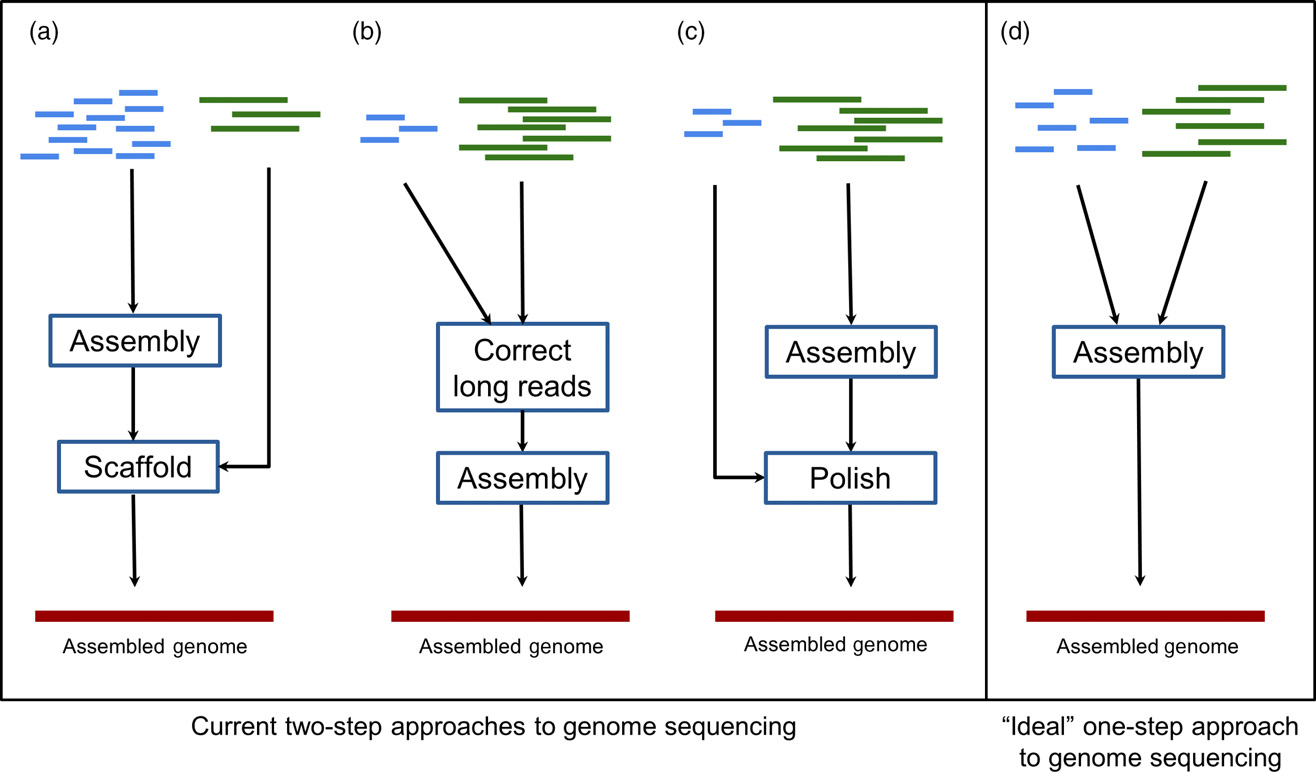
Approaches to genome sequencing. Currently, when approaching genome sequencing, the method used depends on the read lengths available: (a) When more short reads are available, they are first assembled into contigs, which are then scaffolded, guided by the long reads. When more long reads are available, two assembly options exist. Either (b) short reads are used to first correct the long reads, which are then assembled or (c) the long reads are first assembled after which the short reads are used to ‘polish’ the assembly. As these approaches lose information at each step, a method (d) that could combine long and short reads in a single step (theoretically leading to an improved genome assembly) would be optimal.

Given their complementary attributes, it is common to combine error‐prone long reads with highly accurate short reads to form a potentially superior hybrid assembly (Figure 2). Multi‐step hybrid approaches are necessary because established assembly algorithms, namely the Overlap‐Layout‐Consensus (OLC) method, used with long reads, and the De Bruijn Graph (DBG) method, used with short reads, are only suitable for their respective kinds of read dataset. An early approach to hybrid assembly was to first assemble the short reads, then scaffold the resulting contigs guided by the long reads (Figure 2). This process can be performed by a post‐assembler tool, such as PBJelly and SSPACE‐LongRead, or by integration directly into an assembler, such as SPAdes. The MaSuRCA (Zimin *et al*., 2013) approach is similar and works by first conservatively assembling the short reads into longer ‘super‐reads’ and then assembling the super‐reads in combination with the longer reads in an OLC approach. These short‐read‐first approaches work relatively well when only limited amounts of long‐read data are available.

However, when sufficient long‐read data are available, a long‐read assembly approach will generally give a better result. Short reads can be used before assembly to correct the individual long reads (Figure 2b) or after assembly to correct the contigs (Figure 2c), a process commonly referred to as ‘polishing’ the assembly. For pre‐assembly read correction, the simplest approach is to map individual short reads onto long reads and use the short‐read consensus to correct the long reads. This approach is implemented in tools such as Proovread (Hackl *et al*., 2014) and/or LSC (Au *et al*., 2012). As it is difficult to unambiguously align individual short reads against long reads, an alternative strategy involves an initial assembly of the short, accurate reads into contigs (HALC, Bao and Lan, 2017) or assembly graphs (LoRDEC, Salmela and Rivals, 2014) to correct the long reads. A recent comparison of long‐read correction tools found that HALC performed best on data sets from ‘complex’ genomes, such as that of humans or rice (Mahmoud *et al*., 2017).

Post‐assembly polishing using short reads can be performed using Pilon, while Racon supports polishing with either short or long reads. Although polishing with accurate short reads can dramatically improve assembly accuracy, in practice, this often applies only to unique genome regions.

Although these multistep hybrid approaches often outperform assemblers, which use short or long reads alone, they are inherently wasteful. Information is lost at each step in the analysis, and therefore results in a suboptimal assembly. A single step hybrid approach, which would allow for the seamless integration and combined analysis of short and long reads, could in principle yield an improved assembly (Figure 2d).

In addition, especially for some plant genomes, many short reads cannot be accurately mapped to one location due to transposon‐derived repeats and homologous genes with a high degree of identity, making the long‐read assembly errors unrecoverable by short reads.

The final endpoint of a genome assembly is ordering and orienting the assembled sequences to form chromosomal pseudomolecules. This can be guided by marker sequences from an independently determined genetic map. Alignment of these marker sequences against the assembly allows the approximate chromosomal position and potentially orientation of each scaffold to be determined. This last step is often not reached, as it is either not needed for the planned analyses or high resolution genetic maps are not available. However, in the context of combining genotypes with phenotypes, the exact chromosomal position of genes is essential for their correlation with known QTLs. Hi‐C, a new technology providing contact frequencies between sequences, has revolutionized the assembly to chromosomes. For plants it was notably applied to the 5 Gb barley and 12 Gb wild emmer genome (Avni *et al*., 2017; Mascher *et al*., 2017) and has allowed chromosome‐scale assemblies without a genetic map for example for raspberry (Van Buren *et al*., 2018).

### One reference, multiple references and the pangenome

Short‐read sequencing technologies, in conjunction with annotated reference genomes, can be readily applied to a variety of biological questions, including detection (Zhang *et al*., 2017) and analysis of gene expression (Ezer *et al*., 2017), DNA methylation (Zhong *et al*., 2013), identification of transcription factor binding sites and the detection of causal regions and mutations in mutant screens (James *et al*., 2013; Klap *et al*., 2017) or populations (Thoen *et al*., 2017). However, the importance of next‐generation sequencing beyond the context of a single reference accession has long been recognized (Varshney *et al*., 2009). As sequencing became more accessible in terms of cost and availability, plant projects frequently sequenced multiple accessions or species in order to investigate natural diversity. This was initially applied to plant species with relatively small genomes such as rice or Arabidopsis, but has since been extended to field crops such as tomato (Lin *et al*., 2014).

Traditionally, the dominant analysis approach for such projects involved mapping reads from novel accessions to the reference genome to determine small‐scale variation, especially single‐nucleotide polymorphisms (SNPs) and, less commonly, insertions and deletions (InDels) including copy‐number variations (CNVs). A reduced representation of a genome is potentially the cheapest way to gain SNP and marker information in order to enable genome‐wide association studies (GWAS) and genomic selection studies (Bhat *et al*., 2016). The key idea was to reduce the sequencing cost per sample by only sequencing corresponding parts of genomes, albeit at the cost of a more complex library preparation, using restriction enzymes to selectively cut the DNA, therefore focusing the sequencing around the restriction sites. Multiple approaches have been developed, including reduced representation libraries (RRL; Van Tassell *et al*., 2008), restriction site associated DNA sequencing (RAD‐Seq; Baird *et al*., 2008) and genotyping‐by‐sequencing (GBS; Elshire *et al*., 2011). New variations of these techniques continue to be developed (He *et al*., 2014; Scheben *et al*., 2017).

Whole genome resequencing ranging from skim sequencing (approximately 1× coverage or below) to medium coverage resequencing (in the range of 20–40×) is increasingly common for small to mid‐size genomes (Scheben *et al*., 2017), but remains so far prohibitively expensive for large genomes such as wheat. Using this resequencing approach, sequences from the whole genome are used, thereby offering more comprehensive SNP detection than reduced representation approaches. This whole genome resequencing approach, which was successfully used in humans, often performs less well when applied to plants. This is due to the standard read mapping approaches, which were mostly tuned for human data sets and only tolerate minor variations from the reference sequence (Li and Durbin, 2009; Langmead and Salzberg, 2012). These are therefore ill suited to the high rates of variation found even within a single plant species. The frequent use of related wild species as breeding material further amplifies this problem, due to a broadening of the genomic pool. Other typical plant genomic characteristics, including large gene families, ancient whole genome duplications, polyploidy and a high amount of transposon derived repeats, further exacerbate the challenge.

While techniques based on mapping reads to reference genomes are well suited to GWAS and genomic selection, they are inadequate in identifying new genome variants, such as novel genes not present in the reference. In maize, it was estimated that an early genomic reference did not capture about a quarter of the low‐copy gene fraction from all inbred lines (Gore *et al*., 2009). Despite the estimated completeness of this reference being just 91%, mapping rates of above 95% for whole genome resequencing were obtained, illustrating that some reads were incorrectly mapped to repeat regions or paralogous genes (Bukowski *et al*., 2018). This represents a major issue as, in order to improve existing elite accessions using transgenic and new breeding technologies, finding novel genes or gene variants is necessary (Scheben and Edwards, 2018).

An alternative strategy to deal with this issue is to map the reads to the reference plant genome using relatively strict alignment criteria, followed by assembly of the ‘left‐over’ reads that could not be mapped. Using this two‐step approach, it is expected that the non‐mapping reads will assemble into novel genetic regions present in the particular strain under study. This strategy has been applied in the model plant Arabidopsis (Schneeberger *et al*., 2011), and more recently to crops cabbage (Golicz *et al*., 2016) and wheat (Montenegro *et al*., 2017). However, the resulting novel sequences are typically short and fragmented, as many of the reads belonging to these regions would have been inadvertently mapped to similar regions present on the reference, even if relatively strict alignment criteria are used.

A radically different approach is to ignore the existing reference entirely, instead jointly assembling read data from multiple genomes and tracking read origin (Iqabl *et al*., 2012, Muggli *et al*., 2017). This computationally elegant method allows the nodes and/or edges of the graph to be tagged with information, indicating which read dataset(s) support them. Given these tags, it is easy to determine the nodes/edges that are either shared by or unique to specific datasets. Chains of such nodes/edges can then be used to infer longer shared or unique sequences. Despite its elegance, this approach is only used occasionally in the eukaryotic field due to the computational resources needed.

Another alternative is the creation of multiple *de novo* assemblies that, from the wet‐lab perspective, has been made feasible by recent advances in long‐read sequencing technologies. However, the bioinformatics infrastructure required for such an endeavor presents a major barrier. A single gigabase scale assembly can require 10 000+ CPU h per iteration for Canu (Koren *et al*., 2017, Schmidt *et al*., 2017), but new sequence analysis algorithms (Bolger *et al*., 2017b,c) and assembly tools such as wtdbg (see above) promise to bring these computational costs down.

Multiple *de novo* genomes from a single species contain a more complete genetic repertoire than a single haploid reference. This approach can be extended to a set of related species such as a crop and its wild relatives. A recent study used more than 60 diverse rice (*Oryza sativa*) accessions together with a wild relative (*Oryza rufipogon*) to assemble multiple genomes, revealing gene loss and gain (Zhao *et al*., 2018). In a similar approach, 54 *Brachypodium* lines were all assembled *de novo* (Gordon *et al*., 2017). While illustrating the power of a multiple‐reference genome approach, these projects required multiple time‐intensive analysis pipelines, and several *ad‐hoc* developments.

A fundamental barrier to the wider adoption of this approach is that the vast majority of existing analysis tools and pipelines do not work with multiple‐reference genomes. The naïve creation of an *in silico* polyploid, formed by aggregating multiple‐reference genomes, is inadequate in many scenarios. It is necessary to have a clear conceptual difference between the sequences from a single line/species, which are generally considered in aggregate, and sequences from different lines/species, which are considered as alternatives. Furthermore, this *in silico* polyploid approach is highly inefficient when working with a large number of highly related genomes, as each is represented independently.

The creation of the pangenome promises to address the conceptual and computational limitations of the *in silico* polyploid approach. At its most basic, the pangenome must retain the distinction between multiple sequences from one origin genotype and sequences from different genotypes and, more critically, the analysis tools using the pangenome reference must act appropriately based on this origin information and the specific analysis being performed. For computational reasons, the pangenome is likely to be represented as a graph structure, as described above, rather than a large collection of independent linear sequences. This allows regions that are shared between many genomes to be represented once, saving both storage space and computational resources during alignment.

Despite the challenges of their creation, pangenomes promise to be an extremely powerful resource for analysis of genomic sequences. However, existing pangenomic aligners, such as BWBBLE (Huang *et al*., 2013) can handle only limited variation beyond what is already known. This limitation is not critical for genomes (e.g. human) in which genetic diversity is limited and in which the reference is very comprehensive. However, for optimal use with crop species and their wild relatives, pangenomic tools will also need to support highly divergent, novel sequences as well as large‐scale variations. One approach has been made by the Variant Graph Team (Variant Graph Team, 2018) that allows the representation of pangenomes in graphs or to map reads to these and also to visualize them.

In summary, using multiple‐reference genomes, it is possible to find new genes or new regulatory *cis* elements. This would not be possible with only one reference. Especially in the case of regulatory elements, line‐specific transposon insertions bringing their own regulatory elements might play an important role (Chuong *et al*., 2017).

### Standardized genome annotations

**Figure 3:**
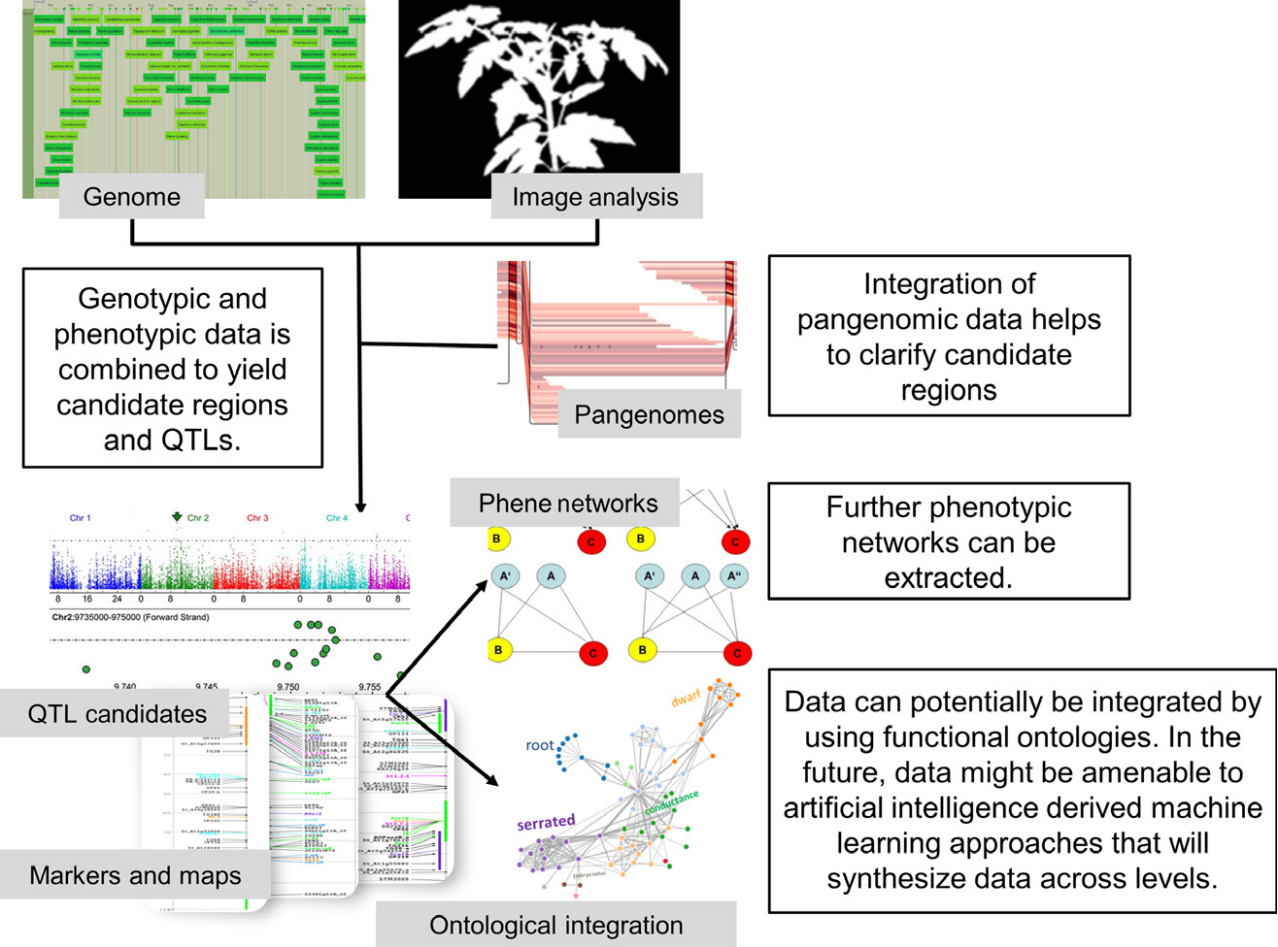
Combining genomic and phenomic data. The GWAS image was taken from Voiniciuc *et al*. (2016).

To find and functionally annotate causal genes that underlie a QTL region, it is first necessary to identify these genes in the underlying DNA sequences (Figures 1 and 3, left panel). While gene finding can still be considered as an art, tools such as MAKER‐P (Campbell *et al*., 2014) and BRAKER2 (Hoff *et al*., 2016) have simplified this task considerably. In situations in which sufficient RNA‐Seq expression data are available, programs such as StringTie (Pertea *et al*., 2015) can be used to transform these data into a first draft gene space. This expression‐driven gene calling improves with the use of full‐length cDNA sequencing, made possible by long‐read technologies. However, expression‐driven gene annotation can only detect genes for which a data set exists and in which these genes are expressed. This necessitates that samples are subjected to a wide range of conditions to activate expression of the full gene space.

In comparison to gene finding, a comprehensive transposon detection method for plant genomes is still in a more experimental phase. There are currently no established pipelines that capture all transposon types in a single step. This does not pose a major problem when working with a new genome for which a well curated repeat library exists from closely related species. In such cases, a simple homology search against repeat libraries provided by repeat databases such as RepBase (Bao *et al*., 2015) or PGSB‐REdat (Spannagl *et al*., 2017) will be sufficient to provide a draft transposon annotation. Suitable matching tools are either RepeatMasker (Smit *et al*., 2016) or vmatch (http://www.vmatch.de/), which greatly improves running times for large genomes (Avni *et al*., 2017; Mascher *et al*., 2017). For novel species without curated repeat libraries, the transposon annotation is more cumbersome as *de novo* detection of species‐specific transposons needs to be performed first (Lerat, 2010). Here, packages like REPET (Flutre *et al*., 2011) perform well for smaller genomes. Transposons, formerly considered as junk DNA, are now believed to be a major contributor to genotype diversity. Their role in phenotype diversity has been shown for many well studied single examples (e.g. Butelli *et al*., 2012; Lutz *et al*., 2015) and also in some genome‐wide approaches (e.g. Bolger *et al*., 2014a,c; Makarevitch *et al*., 2015). Given the emerging importance of transposons in the study of stress and developmental responses, their consistent annotation and analysis is crucial and will be likely to provide many interesting insights and, when pangenomes are available, allow tracking of transposon evolution in a species.

Once genes and transposons have been structurally annotated, the next step is to ascribe each gene a biological function, in a process known as ‘functional annotation’. While using one‐off textual annotations can be beneficial when inspecting small QTL regions for potential candidates, using *a priori* biological knowledge is no longer feasible for large‐scale analyses. Therefore, a full genome annotation will usually first rely on an automatic functional annotation based on domain analyses and sequence similarity searches. In order to provide consistency, most tools that automatically annotate genomes frequently employ formalized ontologies such as Gene Ontology (GO) or MapMan ontology (Jaiswal and Usadel, 2017). The use of these (or other well defined) ontologies enables consistency of the annotation terms between different genomes.

There are many tools available that automatically annotate genes using ontologies such as the Mercator automated annotation tool (Lohse *et al*., 2014), BLAST2GO (Conesa and Gotz, 2008), KEGG Automatic Annotation Server (KAAS) (Moriya *et al*., 2007), and TRAPID (Van Bel *et al*., 2013) (reviewed in Bolger *et al*., 2017a). The overarching goal of these tools is the rapid automatic annotation of genes to a high standard, approaching that of manual annotation.

### Phenotypes and their standardization

An important goal of plant genomics and other ’omics approaches is to better understand and predict plant phenotypes. Despite the challenges involved in plant genomics research, the generation and analysis of genomic data are largely outpacing the production and interpretation of phenotyping data (Furbank and Tester, 2011; Cobb *et al*., 2013). The reason for this ‘phenotyping bottleneck’ is that plants are highly plastic; one genotype may exhibit many different phenotypes depending on environmental conditions. Considerable efforts have been invested into the automation of plant phenotyping (Fiorani and Schurr, 2013; Fahlgren *et al*., 2015; Shakoor *et al*., 2017), which has dramatically improved the consistency and throughput of plant phenotyping.

However, even more than in genomics or other ’omics disciplines, plant phenotyping is a multi‐dimensional challenge, especially for crop species. This is because complex, commercially important targets, such as ‘yield improvement’, result from a variety of physiological, morphological, anatomical, and chemical aspects of plant performance. Therefore, many phenotyping efforts aim to understand one or more of these components such as photosynthesis, root architecture, or above ground biomass and subsequently build on crop models to scale to yield (Parent and Tardieu, 2014).

Given the developmental changes observed over time from seedling to mature plant, emphasis of most newly developed phenotyping techniques is on non‐destructive approaches, such as (three‐dimensional) imaging with red, green and blue (RGB) cameras (Figure 1), thermal and hyperspectral imaging, and/or fluorescence measurements of photosynthesis. Analyses of physiological processes such as enzyme activities (Gibon and Rolin, 2012), transpiration or carbon flux (carbon gain through photosynthesis, carbon loss through leaf, stem and root respiration) are far more challenging, as automation in these fields is not straightforward. Automated sampling of leaf material using robots will represent an important advance (Alenyà *et al*., 2012).

A single genotype has the potential to display a range of different phenotypes depending on the environmental conditions to which it is subjected. One challenge for researchers is to consider and address logistical issues that arise when coordinating physiological studies. For example large, in‐depth studies (such as for GWAS and QTL analyses) require considerable experimental space and resources necessary for the growth and analysis of a wide array (>200) of plant genotypes. Proper attention must be given to environmental conditions, ensuring that they are consistent across all replicates. Constant environmental conditions allow for a better assessment of physiological responses and most analyses are typically carried out with plants that are grown individually in pots, either in a growth room with small plants such as Arabidopsis, or in the glasshouse with larger, but agriculturally more relevant species such as *Triticum* or *Zea*. Small pot sizes ensure that there is enough space for many replicates as well as easy handling in automated phenotyping stations, but may also limit plant growth (Poorter *et al*., 2012a). Environmental conditions are generally under tight control in growth rooms and, to a lesser extent, in glasshouses. Nevertheless, both growth room and glasshouse environments are significantly more stable than the fluctuating environmental conditions that plants experience when subjected to field conditions. Consequently, genotypes that perform well in controlled environments may not necessarily be the ones that perform best in the field. Care has therefore to be taken when choosing and testing relevant conditions, i.e. light and temperature (Poorter *et al*., 2016). This is especially true when plants are tested under suboptimal conditions, such as a low nutrient or water supply (Ingestad, 1987). Limiting pot size, or improper timing of induced stresses could make the entire phenotypic analysis irrelevant (Passioura, 2012). Finally, when plant performance in the field is the ultimate aim, one has to keep in mind that a genotype that thrives well when grown individually in a pot may not necessarily be the genotype that will perform best under conditions in which plants are grown at high densities, such as in agriculture (Tollenaar and Wu, 1999).

Given the important role that the environment plays in plant growth and development, a comprehensive report of environmental conditions during experiments is of paramount importance, both for experiments carried out under controlled conditions as well as in the field (Poorter *et al*., 2012b). This enables the comparison of outputs of various experiments and to develop the ideotypes for different environmental scenarios (Chenu *et al*., 2011). Additionally, improved data sharing and standardization in reporting, particularly with regard to phenotype responses, is especially important in the agricultural sciences (Zamir, 2013). Making historic phenotypic data publicly available would allow plant researchers to share results, compare phenotypes, and analyze data that has been deposited in the past in order to identify new, and sometimes rare, alleles that improve productivity. Finally, the low‐barrier accessibility of data would invite computer scientists and computational biologists to develop or improve current algorithms for phenotypic data analysis (Minervini *et al*., 2016) and support the integration of scientific fields.

Although this is not always easy given the wide array of plant traits that are measured and the specific developmental time points during which those data are collected, efforts on data standardization are rapidly improving (Figure 1) (Krajewski *et al*., 2015; Ćwiek‐Kupczyńska *et al*., 2016). This will undoubtedly facilitate broader application of techniques such as genome‐wide association studies (GWAS; Millet *et al*., 2016) using high‐throughput field phenotyping (Pauli *et al*., 2016) (Figure 3). However, it is also important to keep in mind that there is not only a need for advancing phenotypic analysis and data integration, but also for better insights into the application of knowledge obtained under controlled conditions for the improvement of plant performance in the field (Junker *et al*., 2014; Poorter *et al*., 2016).

### Phenotypic data storage

One key challenge in the plant sciences is the definition of appropriate data management procedures and infrastructures to preserve research data as a valuable scientific asset. This task has been centralized for genomic and expression data for all fields of the life sciences with the Short Read Archive (in the USA) and European Nucleotide Archive (in Europe). Phenotypic data, due to its high divergence, cannot easily be tackled by a highly streamlined and generalized platform. However, in line with the value of original data, funding agencies (Mons *et al*., 2017) and scientific journals are increasingly requesting scientists to publish research data under the FAIR (findable, accessible, interoperable, and reusable) data principles (Wilkinson *et al*., 2016). To make data reusable and interoperable in the plant phenotyping community, MIAPPE recommendations (i.e. required Minimal Information about Plant Phenotyping Experiments) are being developed to ensure a proper description of all necessary metadata, including the environment (Krajewski *et al*., 2015; Ćwiek‐Kupczyńska *et al*., 2016).

Nonetheless, complex, heterogeneous, or unstructured research data frequently remain publicly unavailable, often due to the lack of infrastructure needed to handle these data. In other cases, the data are published but remain obscured within the supplementary materials. While such data are human interpretable, the lack of standardized formatting and data semantics makes automated approaches difficult and error prone.

To provide a generalized resource with an emphasis on phenotypic data, the FAIR‐aware e!DAL software library was developed. Its aim was to lower the technical barriers and minimize the effort of researchers to make data publicly available (Arend *et al*., 2014). By contrast with popular data publication platforms such as Figshare (Singh, 2011) or DRYAD (White *et al*., 2008), e!DAL enables access to large volume research data stored in‐house by assigning Digital Object Identifiers (DOIs). While Figshare and DRYAD offer a comprehensive functionality, they are only free up to a relatively low data volume. This makes them an ideal solution for sharing condensed tables or reduced figures but these resources quickly become expensive and time consuming for larger phenotypic datasets. While there are other generic data repository infrastructure libraries available, for example Fedora (Lagoze *et al*., 2006), they do not provide a ready‐to‐use implementation as within e!DAL. Based on e!DAL, the Leibniz Institute of Plant Genetics and Crop Plant Research (IPK) Gatersleben and the German Plant Phenotyping Network jointly initiated the Plant Genomics and Phenomics Research Data Repository (PGP) (Arend *et al*., 2016a), which provides amongst others the first full MIAPPE compliant (Ćwiek‐Kupczyńska *et al*., 2016) phenotypic datasets (Arend *et al*., 2016b; Chen *et al*., 2018). The PGP repository currently provides 150 data records linked by DOIs and annotated by technical metadata. This comprises more than 1.2 million files with a volume of over two terabytes and is coupled to the ELIXIR European bioinformatics infrastructure, which allows a single sign‐on service. Furthermore, another unique feature of e!DAL‐PGP is the integrated peer‐review process, which guarantees a certain data quality for every released dataset. The intuitive submission process supports researchers in describing and sharing their phenotypic data to exploit the full scientific potential of their data.

The MIAPPE compliant form of data storage promises to overcome standardization issues especially for experimental factors, as discussed in the previous section. Therefore, these datasets will be immediately useful for experimental reproduction or offer a secondary use. Once enough data have accumulated they can be mined from different databases or e!DAL installations using for example DOIs and potentially identify relevant datasets by MIAPPE tags, offering a true multi‐player international data structure. This allows large‐scale data producers to share their data without a centralized resource by relying on existing infrastructure. Afterwards, the collective dataset might be subjected to machine‐learning approaches, discussed below.

However, to profit from existing phenotypic data straight away, it is potentially useful to simplify the environment to a single factor, such as water availability (see above and Poorter *et al*., 2012b) and the unit of measurement to a simple (ontological) term (e.g. ‘days to flowering’). A similar approach focusing on phenotypes is chosen by the AraPheno database, which collects several hundred phenotypes for the model plant Arabidopsis (Seren *et al*., 2017), many of which are derived from one large‐scale study by a multiauthor group (Atwell *et al*., 2010).

Due to the knowledge about the underlying populations, the data can be transmitted into a standardized GWAS pipeline in AraGWAS, which relies on standardized statistics and will therefore offer more comparable results (Togninalli *et al*., 2018).

Finally, it can be useful to store and summarize data even more simply, i.e. to only keep data relating to QTL for a specific species (Nijveen *et al*., 2017) or a group of species (Ni *et al*., 2009), as this provides, at the very least, a way to compare between different analyses and means to confirm results when a plant researcher or breeder conducts a similar analysis.

### Bridging genotypes and phenotypes

Associating genotypes and phenotypes has become much more simple, as statistics have matured and state‐of‐the‐art tools that can be used on a user's desktop to associate data, for example in GWAS‐type settings (Figure 3), have been developed. These tools range from the efficient mixed model (EMMA) type family, through FAST‐LMM (Lippert *et al*., 2011) to TASSEL (Bradbury *et al*., 2007), to name but a few that are reviewed in this issue.

Additionally, user‐friendly online tools such easyGWAS (Grimm *et al*., 2017) or GWAPP (Seren *et al*., 2012) exist. These tools only require phenotypic data if using Arabidopsis. This is because these tools analyze the phenotypic data against an internally stored set of genomic data from a reference panel.

However, high‐throughput phenotyping of multiple traits allows not only association of traits with genotypes, but also association of traits with each other (e.g. Poorter *et al*., 2014, Figure 3). Once again, this works best within the same experimental setting, as under these conditions the environment and management is by definition ‘identical’. However, it is clear that novel insights would require pooling of multiple datasets or very large datasets that comprise many different phenotypic values; this has been done in AraPheno/AraGWAS (see above).

Another large advantage of an approach that relates phenotypes to phenotypes is that comparatively few variables are concerned, making statistical overfitting a minor problem. This advantage is because phenotypic data (on large populations) does not suffer from ‘p≫n’, i.e. the number of variables (phenotypes, p) is usually not larger than the number of samples (n). As an example, the Atwell study (2010) recorded 107 diverse phenotypic values in between 90 and 180 accessions or more. Therefore, many techniques from the extensively studied field of gene network reconstruction (reviewed by Emamjomeh *et al*., 2017) work well if not better when applied to phenotypes, given a large enough population. Indeed, for plant gene network analyses, gene expression data are often simply correlated, without putting too much detail into environmental or perturbation conditions. The only consideration is that expression data sets should represent a range of different conditions and not favor certain perturbations over others. This could be done either by hand, for example in CSBDB.DB (Steinhauser *et al*., 2004), or automatically, for example in ATTED‐II (Obayashi *et al*., 2018).

However, while a simple correlation analysis between phenotypes is a good start for an analysis and therefore supported in AraGWAS (Togninalli *et al*., 2018) and Phenome‐networks, more sophisticated approaches can be used. Indeed multiple different statistical and machine‐learning approaches are already being used currently.

Firstly, as a way to bridge for example well refined molecular measurements such as metabolic profiles to physiological parameters, one can use partial least square (PLS). This technique allows the determination of relationships between outcome variables and predictor variables. Gago *et al*. (2017) used this approach to relate canopy and stomatal conductance from a vineyard to a metabolite matrix. Typically, PLS results are then analyzed using variable importance prediction to determine important predictors (i.e. metabolites in this case). Gago *et al*. (2017) found for example phenylpropanoids and *myo*‐inositol to be predictive for both conductance values.

Alternatively, machine learning can be employed to predict important factors such as biomass. As an example, Maddison *et al*. (2017) used classical machine‐learning techniques (feature selection coupled to support vector regression) to predict biomass outcomes from non‐structural carbohydrates in *Miscanthus*, extending earlier observations by Sulpice and colleagues in Arabidopsis (2013).

However, these approaches implied that certain variables are considered *a priori* as more important than others. While this is clearly the case for *Miscanthus* biomass, deep phenotypic data allow the uncovering of novel associations not observed previously between the individual variables.

The QTL + phenotype supervised orientation (QPSO) approach, developed in the van Eeuwijk laboratory (Wang and van Eeuwijk, 2014; Wang *et al*., 2015), aims to generate directed networks between phenotypic traits by using known sparse QTL to orient the network, extending earlier work on gene network reconstruction and cleverly combining different data domains.

However, when only phenotypes are concerned, one can consider (full) partial correlation analysis that removes the influence of other variables on a variable pair or Bayesian network reconstruction. Common to all these methods is that they try to find relationships between two entities that are not dependent on the other variables. As an example, consider abscisic acid (ABA) that influences both stomatal conductance (Wilkinson and Davies, 2002) and primary root growth (Rowe *et al*., 2016) in response to drought stress. Assuming an overly simplified model in which stomatal conductance and primary root growth were only dependent on the ABA concentration, all three items would be correlated. However, controlling statistically for ABA would reveal that stomatal conductance and primary root growth were unrelated.

In any case, none of these takes hidden (not measured) but potentially important and causal variables into account. In addition, while these methods do not link traits or physiological variables with the underlying genomic basis (except for QPSO), they do provide structural insights about trait interrelationships. This understanding can be used to modify a target trait by genetically modifying another trait whose genetic basis is already understood. However, it has to be noted that all modelling insights are restricted to the data at hand, meaning many missing variables will make this more difficult.

### Phenotypic prediction using phenotype ontologies

Another valid abstraction approach is to couple phenotypes to genes or genomic regions, leveraging a meaningful phenotypic ontology (Zamir, 2013, Hoehndorf *et al*., 2015; Deans *et al*., 2015; Coppens *et al*., 2017, Figure 3 top left). This strategy has been employed for many years for animals and humans, reaching from phenotypically described and formalized mouse data to integrated environments and reasoning, bridging data from different species (Robinson and Webber, 2014; Mungall *et al*., 2017; Rodríguez‐García *et al*., 2017). These data being animal−human centric are centered around disease associations, however the plant community has (at least for Arabidopsis) a massive resource for single knockouts using T‐DNA lines (O'Malley and Ecker, 2010; Kleinboelting *et al*., 2017). As a result, many ontologically defined phenotypic annotations are already available for knockouts and other transgenics in The Arabidopsis Information Resource (TAIR) and other databases (Lloyd and Meinke, 2012; Akiyama *et al*., 2014).

Therefore, data on phenotypes resulting from knockouts could be integrated with GWAS studies using the phenotype ontology data integration framework developed by the animal community (Hoehndorf *et al*., 2015). Therefore, typical candidate approaches, in which genes underlying a QTL region are investigated manually, could be extended by selecting candidate genes, based on their phenotypes and/or based on where in a phenotype network they reside. Indeed, the Planteome project tries to assess and integrate some of these data already with clever use of biomedical ontologies (Cooper *et al*., 2018).

### The blessings and curses of machine learning

Over the past few years ‘deep’ machine‐learning methods, and particularly artificial neural network‐based approaches, have led to revolutionary results, particularly in image analysis. For example, this has greatly spurred the identification of plant features such as root tips and their localization in an image (Pound *et al*., 2017) to count leaves (Ubbens *et al*., 2018) or to derive vegetation indices from RGB images (Khan *et al*., 2018). In addition, this has led to the development of methods to detect plant diseases (Mahlein, 2016; Mohanty *et al*., 2016; Fuentes *et al*., 2017) and plant stress phenotyping (Ghosal *et al*., 2018). The latter application of deep learning to plant abiotic and biotic stress phenotyping has recently been reviewed by Singh *et al*. (2018).

The underlying frameworks are constantly driven forward by Google, Facebook, and other companies offering readily usable frameworks such as Tensorflow (https://www.tensorflow.org/) or Caffe2 (https://caffe2.ai/). In addition these big data centered companies develop dedicated hardware that promises to greatly accelerate training and analysis tasks. Therefore it is not surprising that plant image data is analyzed using a plethora of machine‐learning approaches (Pound *et al*., 2014; Tsaftaris *et al*., 2016). However convolutional neural networks have the potential to greatly advance the field of plant image analysis (Pound *et al*., 2017; Ubbens and Stavness, 2017; Figure 1).

One challenge with image analysis is that large‐scale datasets with data and ground truth outcomes are required. The former can be made readily available through plant phenotyping platforms, but finding the ground truth for a limited number of training datasets currently relies mostly on human experts. However, as this is costly and time consuming, smart solutions, such as those relying on citizen science (Giuffrida *et al*., 2018) are needed. A recent clever proof‐of‐concept study, which used the Amazon ‘mechanical turk platform’ (anonymous users are paid for small tasks), performed better than for‐credit students (Zhou *et al*., 2018). Without such data, algorithms can be compared based on standard datasets, such as those supplied by the International Plant Phenotypic Network (Minervini *et al*., 2014). This dataset is suitable for tasks such as plant detection and localization in images, as well as leaf detection, localization, and counting in images. This reliance on training datasets is necessary because there is, as of yet, no application of unsupervised reinforcement learning methods for image analysis purposes.

Other applications of machine learning, such as prediction of plant performance or the integration of heterogeneous datasets, are even less developed as researchers are currently embracing more traditional and/or data science driven methods for these applications. As an example, Chen *et al*. (2018) used regression and random forests, but not deep learning to predict plant biomass from plant images, whereas Coppens *et al*. (2017) reviews data integration.

Plant phenotypic data promise to be interesting vistas for machine‐learning approaches (Figure 3 top). Indeed, early studies have suggested that machine‐learning approaches for phenotype predictions stemming from a sufficiently genotyped population could be meaningful, especially in the p≫n setting in which more predictors from genomics data than plant samples are available (Crossa *et al*., 2017). As an example, Grinberg *et al*. (2018) tried to predict phenotypes using classical genomic Best Linear Unbiased Prediction (BLUP) as well as several machine‐learning techniques. The latter clearly outperformed BLUP for yeast with very controlled environments, whereas for wheat and rice, BLUP performed particularly well when there was population structure (Grinberg *et al*., 2018).

### The non‐model/minor crop plant perspective

As has been shown above, both genomic and phenomic datasets are becoming more and more mature and cost efficient. At this time, it is the model plant Arabidopsis, rather than crop plants, that contains the most extensive datasets and that may enable ontology‐driven phenotype prediction. This is largely due to a number of points: (i) the availability of the machine‐readable ontology term‐enriched phenotypic datasets for well defined genes; (ii) the largest wealth of functional data for gene annotation, which is related to the former point; (iii) the use of standardized populations from the 1001 genome consortium, facilitating abstraction at the phenotypic level; and (iv) standardization, driven for example by TAIR. Also, for genetic and genomic studies, it is necessary to note the importance of accurate phenotyping. The most advanced (in terms of crop plants) is most likely maize that, despite its tremendous genetic variety, is tackled in a well planned and standardized way, driven both (pan)genomically (Gore *et al*., 2009; Hirsch *et al*., 2016) and phenomically (see e.g. AlKhalifah *et al*., 2018; for a well described dataset) and supported by user friendly tools, providing access to these resources such as TASSEL (Bradbury *et al*., 2007). However, while standardization is gaining traction and big datasets are becoming more available for major crops, minor crops remain less supported. Additionally, when studying this genotype−phenotype interaction, it is important to have access to detailed phenotypic data. In many cases, the selection and evaluation of phenotypes have been poorly developed in the experimental design of genetic and genomics studies (Houle *et al*., 2010). Therefore, efforts to identify gold standard experimental procedures and scoring protocols may contribute to the harmonization of phenotypic data and therefore to the improvement of data accessibility (Shrestha *et al*., 2012). In addition, the existence of biases is another new, important challenge in attaining knowledge from new high‐throughput techniques.

That said, for non‐model species general ‘cyberinfrastructures’ can also be used (Merchant *et al*., 2016) and specialized information systems, such as those for grapevine (Adam‐Blondon *et al*., 2016) or the Rosaceae community (Jung *et al*., 2017) have been developed. Indeed, while necessarily less data are available for non‐model (minor) crop plant communities (e.g. an apple researcher); they can learn from the lessons and mistakes made with big crops and within the International Plant Phenotyping Consortium.

Finally, it can be expected that even data from the model plant Arabidopsis will be transferable to dicots (and thus many horticultural minor crops) or at least related crops (i.e. Brassicaceae) on a large‐scale basis going beyond simple gene annotation.

## Conclusions

The impact that the genomics revolution has made on plant science is undeniable and innovative pangenomic approaches that allow the integration of data of related species are beginning to take hold in the plant field. We are therefore in the middle of a genomics data explosion. We are also at an exciting time point, witnessing the next revolution in phenomics (Tardieu *et al*., 2017), and we begin to see how machine learning‐ and data science‐driven approaches are trickling into the area of bridging genomics and phenomics data. These developments are making plant science a truly modern science, inspired by artificial intelligence, robotics systems, and classical plant physiology. A whole new ‘breed’ of quantitative and computer science‐oriented plant scientists (Friesner *et al*., 2017) is therefore required to truly modernize the discipline.

## Conflict of Interest

The authors declare no conflict of interest.

Box 1 **Summary**

- Plant genome sequencing has evolved to soon become a commodity approach for small genomes.
- Phenotypic data standardization recommendations are provided by MIAPPE.
- Many tools and databases facilitate bridging genotypes and phenotypes.

Box 2 **Open questions**

- Algorithms working on multiple genomes of a species are still in development.
- It is still an open question how to best combine short and long reads into assemblies.
- More rigorous phenotype ontologies and machine‐learning approaches are likely to improve our understanding of plants.
